# Flagellin Restricts HIV-1 Infection of Macrophages through Modulation of Viral Entry Receptors and CC Chemokines

**DOI:** 10.3390/v16071063

**Published:** 2024-06-30

**Authors:** Lina Zhou, Xu Wang, Qianhao Xiao, Shazheb Khan, Wen-Zhe Ho

**Affiliations:** Department of Pathology and Laboratory Medicine, Temple University Lewis Katz School of Medicine, Philadelphia, PA 19140, USA

**Keywords:** flagellin, TLR-5, macrophage, HIV-1, CC chemokine

## Abstract

Both bacteria product flagellin and macrophages are implicated in HIV-1 infection/disease progression. However, the impact of their interaction on HIV-1 infection and the associated mechanisms remain to be determined. We thus examined the effect of the flagellins on HIV-1 infection of primary human macrophages. We observed that the pretreatment of macrophages with the flagellins from the different bacteria significantly inhibited HIV-1 infection. The mechanistic investigation showed that the flagellin treatment of macrophages downregulated the major HIV-1 entry receptors (CD4 and CCR5) and upregulated the CC chemokines (MIP-1α, MIP-1β and RANTES), the ligands of CCR5. These effects of the flagellin could be compromised by a toll-like receptor 5 (TLR5) antagonist. Given the important role of flagellin as a vaccine adjuvant in TLR5 activation-mediated immune regulation and in HIV-1 infection of macrophages, future investigations are necessary to determine the in vivo impact of flagellin–TLR5 interaction on macrophage-mediated innate immunity against HIV-1 infection and the effectiveness of flagellin adjuvant-based vaccines studies.

## 1. Introduction

HIV-1 infection disrupts the homeostasis of microbiota in the gut [1,2], and microbial translocation of bacterial products such as flagellin has been associated with HIV-1 disease progression [3,4,5]. Flagellin is the only known agonist of the toll-like receptor 5 (TLR5), and the flagellin—TLR5 interaction is implicated in the systemic inflammation and immune activation during HIV-1 infection, as flagellins can leak into blood circulation through the gut–blood barrier and activate TLR5 on the surface of epithelial cells and the immune cells, particularly in circulation monocytes and macrophages in gut tissues [6]. Therefore, activated monocytes/macrophages are the major source of inflammatory cytokines/chemokines, which contribute to HIV-1 disease progression [2,3]. Clinically, there are increased circulating levels of anti-flagellin antibodies in HIV-1-infected individuals compared to uninfected subjects [7,8,9]. As major immune cells of the host’s first line of defense, monocytes and macrophages can be exposed to translocated bacterial flagellin during viral–bacterial co-infection, which can promote HIV-1 infection through the TLR5 and NF-κB signaling pathways, resulting in the induction of various inflammatory cytokines [6,10]. Therefore, monocytes/macrophages are the key contributors to systemic inflammation and immune activation. Although it is known that flagellin affects the host immunity through its impact on different cell types in the context of viral infections [11,12,13,14], there is limited information about the direct effect of flagellin on HIV-1 infection/replication in macrophages.

Although macrophages are vital for both innate and adaptive immunity against invading pathogens including HIV-1 [15,16,17,18], they are the major target of HIV-1 infection [19]. Macrophages infected with HIV-1 demonstrate increased resistance to apoptosis and decreased sensitivity to combination antiretroviral therapy. These features make macrophages the optimal HIV-1 reservoirs and a key focus for therapeutic intervention [15,16,20,21,22]. An early study [22] reported that TLR5 activation by flagellin could activate NF-ĸB and latent HIV-1 in CD4^+^ T cells in HIV-1-infected individuals. Schlaepfer et al. showed that flagellin treatment reinforced the nonpermissive state of polarized macrophage to HIV-1 infection [23]. However, despite these findings, it is unclear about the mechanism(s) by which flagellin—TLR5 interactions interfere with HIV-1 infection/replication. Given the important role of flagellin as a vaccine adjuvant in TLR5 activation-mediated immune regulation and in HIV-1 infection of macrophages, we examined the impact of the flagellins from different bacteria on HIV-1 infection of primary human macrophages and the mechanism associated with them.

## 2. Materials and Methods

### 2.1. Cells and Reagents

Primary human macrophages were derived from purified peripheral blood monocytes, which were purchased from the Human Immunology Core at the University of Pennsylvania School of Medicine. The Core has Institutional Review Board approval for blood collection from healthy donors who were deidentified. The culture condition for monocyte differentiation into macrophages was described previously [24,25]. Monocytes were plated in 48-well plates (Corning CellBIND Surface, Corning, NY, USA) at the density of 2.5 × 10^5^ cells/well in 0.5 mL complete Dulbecco’s modified Eagle medium (DMEM) with 20% fetal calf serum (FCS), 1% non-essential amino acid, 1% L-glutamine and 1% penicillin–streptomycin solution at 37 °C with 5% CO_2_. On day 7, monocytes differentiate into macrophages (MDMs). Flagellins from three different bacteria (BS: *bacillus subtilis*; ST: *salmonella typhimurium*; and PA: *pseudomonas aeruginosa*) were purchased from InvivoGen (San Diego, CA, USA).

### 2.2. Cytotoxicity Assay

The cytotoxic effect of the flagellins of different bacteria on MDMs was evaluated by MTS (3-(4,5-dimethylthiazol-2-yl)-5-(3carboxymethoxyphenyl)-2-(4-sulfophenyl)-2H-tetrazolium, inner salt) assay. Seven-day cultured MDMs in 96-well plates (10^5^ cells/well) were treated with the flagellins at different concentrations (0 to 1000 ng/mL) for 72 h. The cells were then incubated with CellTiter 96^®^ Aqueous One Solution Reagent (Promega Corporation, Madison, WI, USA) containing MTS and phenazine ethosulfate at 37 °C for 4 h. Absorbance exited at 490 nm was measured and recorded by a plate reader (SpectraMax i3, Molecular Devices, Sunnyvale, CA, USA). As shown in Supplementary Figure S1, flagellin had little effect on the cell viability even at the highest concentration of 1000 ng/mL.

### 2.3. HIV-1 Infection

HIV-1 macrophage-tropic R5 strain (Bal) was obtained from the AIDS Reagent Program at the National Institutes of Health (Bethesda, MD, USA). MDMs (2.5 × 10^5^ cells/well in 48-well plates) were treated with the flagellins (150 ng/mL) of different bacteria for 24 h before HIV-1 Bal (p24 protein 20 ng/10^6^ cells) infection for 2 h. The cell cultures were then washed 3 times to remove the residue of the input viruses and the flagellins. The cells were not treated with flagellins after HIV-1 infection. The culture supernatant was then collected at different time points after HIV-1 infection and subjected to the real-time PCR for the HIV-1 *gag* gene. HIV-1 *gag* standards with known copy numbers were used as the control for quantifying the *gag* gene copy numbers. MDMs were incubated with hTLR5-Fc for 1 h followed by the flagellin treatment for 6 h. The cells were collected and subjected to RNA extraction for the real-time PCR. To investigate the blocking of HIV-1 entry experiments by the flagellins, we used HIV-1 NL4-3-ΔEnv-eGFP-Bal and HIV-1 NL4-3-ΔEnv-eGFP-VSV-G. These viruses were constructed from the HIV-1 Bal Env or VSV-G expression vector with plasmid pNL4-3-ΔEnv-eGFP and provided by Dr. Yun-Tao Wu (George Mason University, USA) [26,27]. The experiments requiring work with infectious HIV-1 in this project were performed in a biosafety level 2 plus (BSL2+) laboratory, and only fully trained personnel were allowed with infectious HIV-1.

### 2.4. RNA Extraction and Reverse Transcription

Total cellular RNA was isolated from MDMs using a Tri Reagent (Sigma-Aldrich, St. Louis, MO, USA). In brief, the total cellular RNA was extracted by a single step, guanidium thiocyanate–phenol–chloroform extraction. After centrifugation at 13,000 g for 15 min at 4 °C, the RNA-containing aqueous phase was collected and precipitated in isopropanol. The RNA precipitates were then washed three times with 75% ethanol and resuspended in 20 μL of RNase-free water. Total RNA (0.5 μg) was subjected to reverse transcription using the reverse transcription system (Promega Corporation, Madison, WI, USA) according to the manufacturer’s protocol. The cDNA was ready to serve as a template for PCR amplification qualification of the chemokines (MIP-1α, MIP-1β and RANTES) and the receptors (CD4 and CCR5).

### 2.5. Chemokine Measurements-qRT-PCR for mRNA

Real-time PCR was performed with the SYBR Green Master Mix (Invitrogen, Waltham, MA, USA). Thermal cycling conditions were designed as follows: initial denaturation at 50 °C for 2 min and 95 °C for 10 min, followed by 40 cycles of 95 °C for 15 s and 60 °C for 1 min. All values were normalized to GAPDH mRNA. The oligonucleotide primers were synthesized by Integrated DNA Technologies, Inc. (Coralville, IA, USA), and sequences will be available upon request. Real-time PCR for the mRNA quantification was conducted as instructed by the manufacturer. The oligonucleotide primers were synthesized by Integrated DNA Technologies, Inc. (Coralville, IA, USA) and sequences are shown in the Table 1 below:

### 2.6. Fluorescent Imaging and Flow Cytometry

MDMs were pretreated with the flagellins (150 ng/mL) for 24 h and infected with VSV-G or Bal-Env pseudotyped HIV-1 eGFP for 72 h. HIV-1 particles in the macrophages were visualized with confocal fluorescent microscopy (Nikon A1R, Nikon, Japan) and processed using ImageJ 1.53c software (NIH, USA). For flow cytometry, MDMs were pretreated with flagellins (BS, ST, PA, 150 ng/mL) for 24 h at 37 °C and detached from the culture wells with Versene solution (0.48 mM EDTA). The harvested cells were washed with cell staining buffer (BD Bioscience, San Jose, CA, USA) containing 0.2% (*w*/*v*) bovine serum albumin (BSA) prior to immunostaining. To detect the cell surface markers expression, MDMs were stained with APC-conjugated anti-CD14 antibody (M5E2, BD Bioscience, San Jose, CA, USA), FITC-conjugated anti-CD4 antibody (RPA-T4, BD Bioscience, San Jose, CA, USA) and PE-conjugated anti-CCR5 antibody (3A9, BD Bioscience, San Jose, CA, USA). Unstained and matched-isotype-stained cells were included as controls. Cells were acquired by FACSCanto II (BD Bioscience, San Jose, CA, USA), and data were analyzed using Flow-Jo V.10.1 software (Tree Star Inc, Ashland, OR, USA).

### 2.7. ELISA

MIP-1α, MIP-1β and RANTES protein levels in MDMs culture supernatant were measured by ELISA kits purchased from the R&D system (Minneapolis, MN, USA). The assays were performed following the manufacturer’s protocols [26].

### 2.8. Statistical Analysis

Data were obtained from three independent experiments and presented as mean ± SD. The variance between the two groups was analyzed by one-way ANOVA by Dunnett’s multiple comparisons test. Calculations were performed with GraphPad Prism 7.0 Statistical Software (GraphPad Software Inc., San Diego, CA, USA). Statistical significance was defined as *p* < 0.05, *p* < 0.01 or *p* < 0.001.

## 3. Results and Discussion

**Figure 1:**
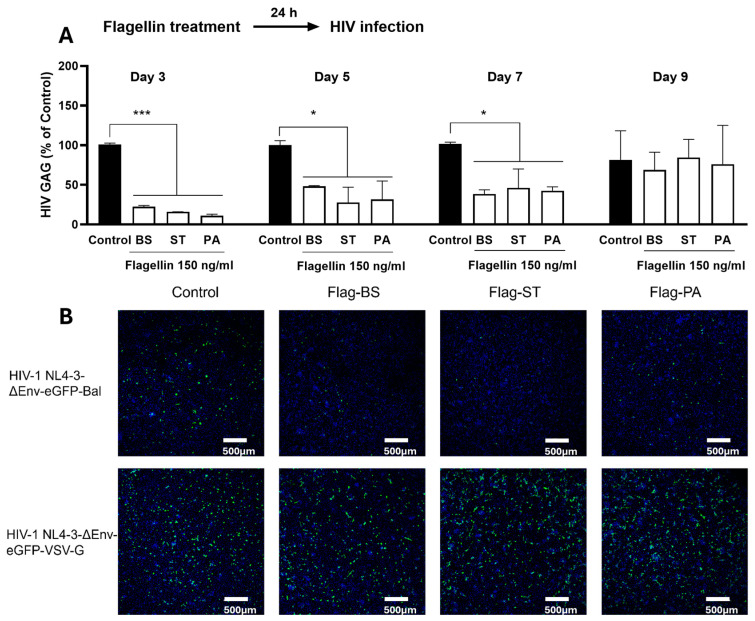
Flagellin blocks HIV-1 infection of primary human monocyte-derived macrophages (MDMs). (**A**) Effect of the flagellins on HIV-1 infection. MDMs were treated with the flagellins of different bacteria (BS: *bacillus subtilis*; ST: *salmonella typhimurium*; and PA: *pseudomonas aeruginosa*) for 24 h before HIV-1 Bal (20 ng/10^6^ cells) infection. Following the 2 h incubation of HIV-1 Bal strain, the cell cultures were then washed 3 times to remove the residue of the input viruses and the flagellins. The cells were not treated with flagellins after HIV-1 infection. The culture supernatant was collected at indicated time points after HIV-1 infection and subjected to the real-time PCR for HIV-1 *gag* gene. Data are expressed as mean ± SD of triplicate cultures, representative of three independent experiments. Data were analyzed with one-way ANOVA (* *p* < 0.05, *** *p* < 0.001). (**B**) Fluorescent imaging of HIV-1 entry to MDMs. MDMs were treated with flagellins for 24 h before HIV-1 infection. The same amount of HIV-1 NL4-3-ΔEnv-eGFP-Bal or VSV-G was added. Following 24 h incubation of HIV-1, the media was replaced with fresh media and infected cells were cultured for 3 days. The cells were then photographed under a confocal microscope (Nikon, A1R, Tokyo, Japan) for green fluorescence measurement. (Scale bar = 500 μm).

Flagellin, a protein essential for bacterial motility, adherence and virulence [23,28], is the only known agonist of TLR5. The activation of TLR5 can induce the intracellular transcription factors, including NF-κB, resulting in inflammatory and other immune responses [29,30,31]. Importantly, the flagellin–TLR5 interaction is implicated in the systemic immune activation during HIV-1 infection, as flagellin can leak into blood circulation through the gut–blood barrier and activate TLR5 on the surface of epithelial cells and the immune cells, particularly macrophages, which are widely distributed in the body [15]. Therefore, it is clinically important to examine the interplays between flagellin and macrophages in terms of HIV-1 infection. We showed that the pretreatment of macrophages with the flagellins from the different bacteria could effectively inhibit HIV-1 infection/replication (Figure 1) without cytotoxicity (Supplementary Figure S1). This finding supports an earlier study by Schlaepfer et al. who showed that flagellin treatment reinforced the nonpermissive state of polarized macrophage to HIV-1 infection [23]. Of note, the flagellin–TLR5 activation-mediated HIV-1 inhibition was potent, as MDMs were only treated once (24 h before infection) with the flagellins at a relatively low dose (150 ng/mL). The significant inhibitory effect of the flagellins on HIV-1 lasted for seven days (Figure 1A). On day 9 after HIV-1 infection, the flagellins showed little effect on the viral replication (Figure 1A), suggesting that the flagellin’s blocking effect was at the early stage of HIV-1 infection.

**Figure 2:**
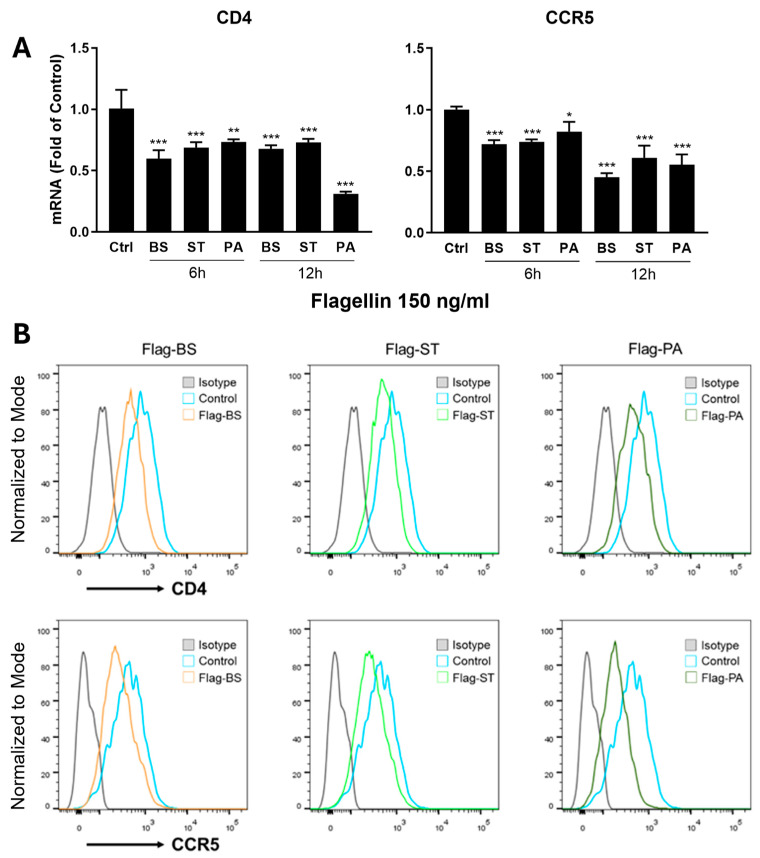
Effect of flagellin on the HIV-1 entry receptors (CD4 and CCR5). MDMs were treated with or without the flagellins from different bacteria (BS: *bacillus subtilis*; ST: *salmonella typhimurium*; and PA: *pseudomonas aeruginosa*). (**A**) Flagellin effect on CD4 and CCR5 mRNA expression in MDMs. The cellular RNA was extracted at 6 h post-treatment and subjected to the real-time RT-PCR for CD4 and CCR5 mRNA. Data are expressed as mean ± SD of triplicate cultures, representative of three independent experiments (* *p* < 0.05, ** *p* < 0.01, *** *p* < 0.001). (**B**) Flagellin effect on CD4 and CCR5 protein expression in MDMs. Flow cytometry analysis of CD4 and CCR5 protein expression at 24 h post-treatment. Cells were acquired by FACSCanto II and data were analyzed using Flow-Jo software.

**Figure 3:**
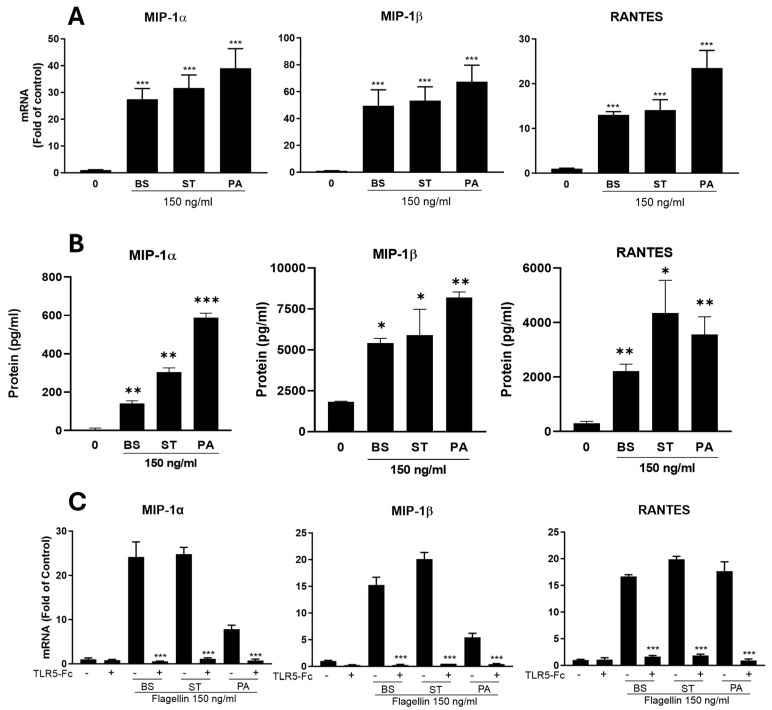
Effect of flagellin–TLR5 on the CC chemokines in monocyte-derived macrophages (MDMs). (**A**) MDMs were treated with or without flagellins from three different bacteria (BS: *bacillus subtilis*; ST: *salmonella typhimurium*; and PA: *pseudomonas aeruginosa*) at the indicated concentration for 12 h. Total RNA extracted from the cells was subjected to the real-time RT-PCR for the mRNA of the indicated CC chemokines (MIP-1α, MIP-1β and RANTES). (**B**) MDMs were treated with or without flagellins from three different bacteria (BS: *bacillus subtilis*; ST: *salmonella typhimurium*; and PA: *pseudomonas aeruginosa*) for 24 h. Supernatants were collected from the cell cultures and subjected to ELISA analysis to measure the protein level of CC chemokines. (**C**) The toll-like 5 receptor antagonist (TLR5-Fc) blocked the flagellin-mediated induction of the CC chemokines. MDMs were incubated with or without hTLR5-Fc (300 ng/mL) for 1 h before the flagellin (150 ng/mL) treatment for 6 h. Total RNA extracted from the cells was subjected to real-time RT-PCR for the mRNA expression of the CC chemokines. Data are expressed as mean ± SD of triplicate cultures and were analyzed with one-way ANOVA (* *p* < 0.05; ** *p* < 0.01, *** *p* < 0.001).

To determine the mechanism(s) of flagellin-mediated HIV-1 inhibition, we examined the impact of the flagellins on two pseudotyped viruses (HIV-1 NL4-3-ΔEnv-eGFP-Bal and HIV-1 NL4-3-ΔEnv-eGFP-VSV-G). Because HIV-1 NL4-3-ΔEnv-eGFP-VSV-G can infect the MDMs without utilizing HIV-1 entry receptors (CD4 and CCR5), the flagellins had little effect on HIV-1 (Figure 1B). In contrast, the flagellins could significantly block infection by HIV-1 NL4-3-ΔEnv-eGFP-Bal (Figure 1B), indicating that flagellin-mediated HIV-1 inhibition occurs at the viral entry level. Our further investigation showed that the flagellin pretreatment of macrophages downregulated the HIV-1 entry receptors (CD4 and CCR5) (Figure 2) and upregulated the expression of the CC chemokines (MIP-1α, MIP-1β and RANTES) (Figure 3A,B), the ligands of CCR5, at both mRNA and protein levels. To determine whether flagellin-mediated CC chemokine induction is specifically through TLR5, we pretreated MDMs with hTLR5-Fc, a soluble antagonist of TLR5, prior to the flagellin treatment. As demonstrated in Figure 3C, the pretreatment of MDMs with hTLR5-Fc could block the expression of the CC chemokines induced by the flagellins. Because flagellin can only be recognized by TLR5, it is unlikely that other PAMPs are directly involved in flagellin/TLR5-mediated HIV-1 infection of macrophages. Taken together, these findings provide the mechanistic evidence for flagellin’s blocking effect on HIV-1 infection of macrophages.

Clinically, flagellin has been used as an adjuvant for the development of vaccines against influenza as well as HIV [14,32,33,34,35]. Therefore, studying the interplays between flagellins and macrophages in terms of HIV-1 infection has clinical relevance and translational value, although further investigations are necessary to determine the in vivo impact of flagellin–TLR5 interaction on macrophage-mediated innate immunity against HIV-1 and the effectiveness of flagellin adjuvant-based vaccines studies. The primary adjuvant effect of flagellin is to induce cytokines and chemokines. Although flagellin is a contributor to systemic inflammation/immune activation, which facilitates HIV-1 disease progression, our data indicate that flagellin appears to be beneficial as an inducer of the macrophage innate immune response against HIV-1 infection. Studies by different groups have shown that flagellin is a monomer protein that can potentially induce both adaptive and innate immunity [36,37,38,39,40,41,42]. Therefore, using flagellin instead of bacteria in HIV-1 research offers the precise activation of specific immune pathways, controlled experimental conditions and reduced biosafety risks. Our studies with the flagellins from different bacteria demonstrate that it is valuable to use flagellin for studies on TLR5-mediated immune responses in macrophages, which can provide not only insights into host cell innate immunity, but also evidence to support the further development of targeted therapies with vaccine adjuvants against HIV-1.

## Figures and Tables

**Table 1:** Primer sets for real-time PCR.

Primer	Accession No.	Orientation	Sequences
GAPDH	NM_002046	Sense	5′-GGTGGTCTCCTCTGACTTCAACA-3′
		Antisense	5′-GTT GCT GTA GCC AAA TTC GTT GT-3′
MIP1-α	NM_002983	Sense	5′-GCTGACTACTTTGAGACGAGC-3′
		Antisense	5′-CCAGTCCATAGAAGAGGTAGC-3′
MIP1-β	NM_002984	Sense	5′-CCAAACCAAAAGAAGCAAGC-3′
		Antisense	5′-AGAAACAGTGACAGTGGACC-3′
RANTES	NM_002985	Sense	5′-CTGCATCTGCCTCCCCATA-5′
		Antisense	5′-GCGGGCAATGTAGGCAAA-3′
CD4	NM_000616	Sense	5′-GCACGACTCTGCAGAAGGAA-3′
		Antisense	5′-CCTAAAAGGGACTCCCCGGT-3′
CCR5	NM_000579	Sense	5′-CAAGTGTCAAGTCCAATCTA-3′
		Antisense	5′-ACCAAAGATGAACACCAGTG-3′

## Data Availability

Dataset available upon request from the authors.

## Supplementary Materials

The following supporting information can be downloaded at: https://www.mdpi.com/article/10.3390/v16071063/s1, Figure S1: The cytotoxicity effect of the flagellins on monocyte-derived macrophages (MDMs). MDMs were treated with the flagellins from three different bacteria (BS: *bacilus subtilis*; ST: *salmonella typhimurium*, and PA: *pseudomonas aeruginosa*) at the indicated concentrations for 72 h. The cell viability was assessed by MTS assays. Data are shown as the absorbance (490 nm).
