# Supplementary figures and images for: Flagellin Restricts HIV-1 Infection of Macrophages through Modulation of Viral Entry Receptors and CC Chemokines
Source: Viruses. 2024 Jun 30;16(7):1063. doi: 10.3390/v16071063 (PMC11281555; doi:10.3390/v16071063)

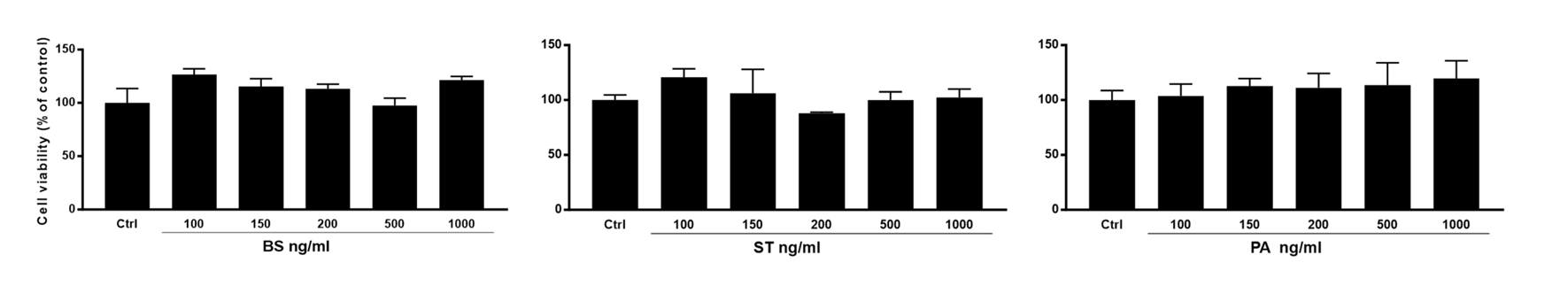

Supplement: Supplementary file 1 [file viruses-16-01063-s001.zip › viruses-3044410-supplementary.tif]
